# Exploration of shared gene signature with development of pre-eclampsia and cervical cancer

**DOI:** 10.3389/fgene.2022.972346

**Published:** 2022-08-17

**Authors:** Tingting Yin, Yin Yin, Lin Qu

**Affiliations:** Department of Obstetrics and Gynecology, The First Affiliated Hospital of Nanjing Medical University, Nanjing, China

**Keywords:** pre-eclampsia, cervical cancer, tumor microenvironment, prognosis, WGCNA

## Abstract

**Background:** The relationship between pre-eclampsia (PE) and cervical cancer (CC) has drawn more attention recently, while little is known about the shared pathogenesis of CC and PE. In the present research, we aimed to generate the shared gene network as well as the prognostic model to reveal the development of CC and PE.

**Methods:** The transcription data of CC and PE patients were obtained and enrolled into weighted gene co-expression network (WGCNA) analysis. Disease-specific modules in CC and PE were determined to discover the shared genes. The expression patterns of genes at protein level were examined by HPA database. Further, LASSO penalty regression and Cox analysis were applied to create a prognostic signature based on the shared genes, with survival curves and ROC plots employed to confirm the predictive capacity. To uncover the function roles and pathways involved in signature, gene set enrichment analysis (GSEA) was conducted. Finally, the immune infiltration status in CC was depicted using CIBERSORT algorithms.

**Results:** WGCNA determined three hub modules between CC and PE. A total of 117 shared genes were obtained for CC and PE and mainly enriched in cell proliferation, regulation of cell development and neuron differentiation. Then, we created a robust prognostic model based on the 10 shared genes by performing stepwise Cox analyses. Our proposed model presented a favorable ability in prognosis forecast and was correlated with the infiltration of immunocytes including B cells, macrophages and T cells. GSEA disclosed that high-risk group was involved in cancer-related pathways.

**Conclusion:** The present project identified the shared genes to uncover the pathogenesis of CC and PE and further proposed and validated a prognostic signature to accurately forecast the clinical outcomes of CC patients.

## Introduction

Pre-eclampsia (PE) is a severe pregnancy-related multifactorial disorder, which can possibly cause the perinatal mortality of the newborn and the mother. Multiple attempts have been made to reveal the pathogenesis underlying PE (Mol et al., 2016; Coviello et al., 2019). Endothelial dysfunction has been considered as a leading cause of PE (Steegers et al., 2010). A number of angiogenic molecules including vascular endothelial growth factor (VEGF), placental growth factor (PlGF), and VEGFR-1 (Flt-1), platelet-derived growth factor (PDGF), and endothelin were reported to alter the angiogenic balance, and further induce the onset and progression of PE (Venkatesha et al., 2006; Wang et al., 2009). Moreover, oxidate stress, which was characterized by lipid peroxidation accumulation, also plays a crucial role in the pathogenesis of PE. Recently, Fragoso et al. reported that the umbilical cords from PE pregnancies showed higher levels of antioxidants including glutathione peroxidase (GPx) and malondialdehyde (MDA) than pregnancies without PE diagnosis, indicating the defensive mechanism to maintain the oxidative balance (Fragoso et al., 2021). Despite all these efforts, the mechanisms regulating the onset and progression of PE remain largely unknown, making it crucial to explore highly sensitive biomarkers to provide an early and intensive care for patients at risk (Grill et al., 2009).

Cervical cancer (CC) ranks as the fourth most frequently diagnosed malignancy in women worldwide (Siegel et al., 2020). Despite the great efforts to diagnosis and treatment, CC still pose a significant burden on women health, especially on those who live in low- and middle-income countries (Hull et al., 2020). The persistent infection of “high risk” genotypes of Human papillomavirus (HPV) is recognized as the most common cause of CC, among which, the most oncogenic HPV 16 and 18 subtypes are accountable for 70% of CC. Briefly, oncoproteins E6 and E7 downregulate the expression level of tumor suppressor genes p53 and pRb, respectively, resulting in genomic instability and a series of aberrant biological phenotypes, eventually leading to tumor initiation (Mittal and Banks, 2017). Conventional strategies for CC treatment mainly including surgical excision, cryosurgery, chemotherapy, and radiation therapy (Gadducci and Cosio, 2020). However, all these strategies can hardly stop the progression, nor the recurrence of CC in the terminal stages, making a comprehensive understanding of biomarkers for CC is urgently needed to guide the future direction of early detection.

Accumulating evidence has pointed out that HPV infection itself cannot explain the carcinogenesis of CC, indicating there exists other biological events involved in the development of cervical lesions (Coker et al., 2002). In this regard, the exploration of additional contributor to the onset and progression of CC is urgently needed (Kreisel et al., 2021). According to Serrand et al., the occurrence of cervical cancer was associated with PE pregnancy history in early life, reflecting the underlying connection between PE and the cancer development (Serrand et al., 2021). It has been hypothesized that the hormone changes brought by PE may alter the homeostasis in reproductive system, which might subsequently influence the incidence of hormone-dependent cancers (Rana et al., 2019; Song et al., 2021a). However, the underlying interaction between CC and PE remain obscure, making a more precise prognosis-related model urgently needed to reveal the similarity of genes that modulate the onset and progression of both CC and PE.

In our current work, a total of 117 genes were identified as “shared genes” for showing similar expression pattern in CC and PE. Based on the “shared genes”, a protein–protein Interaction (PPI) network was constructed with 10 genes being identified as hub genes. Moreover, a prognostic model was established on the basis of the “shared genes”, and the predictive power of the model was analyzed using chemotherapy sensitivity analysis. Finally, we evaluated the patterns of immune infiltration in CC. Our proposed shared genes network reveals the common pathogenesis of CC and PE for the first time, which sheds lights on a deeper understanding in the intrinsic connection between CC and PE. Collectively, comprehensive analyses focusing on shared functional patterns in CC and PE will provide insights into prediction of prognosis and risks, and guide the future therapeutic targets for both diseases.

## Meterials and methods

### Data acquisition

The RNA-seq data and clinical information of CC samples were collected from the TCGA database (https://portal.gdc.cancer.gov/). For PE, GSE60438 containing normalized transcriptome data was downloaded from the GEO website (https://www.ncbi.nlm.nih.gov/geo/) for further investigation.

### Weighted gene co-expression network analysis

“WGCNA” analysis was performed to obtain functional collections by clustering gene into several modules (Langfelder and Horvath, 2008). As described before, the adjacency matrix was employed to capture the connection between the gene pairs using the Pearson correlation analysis. The adjacency matrix was then transformed into a topological overlap matrix as well as the corresponding dissimilarity, and a hierarchical clustering tree was subsequently constructed to show different gene clusters in different colors. Finally, the correlation between the module eigengene and clinical traits were combined to build the co-expression network.

### Construction of PPI network

The shared genes in PE and CC modules with positive Pearson correlation coefficients were overlapped using venn diagram. After that, the STRING online tool (https://cn.string-db.org/) and Cytoscape were employed to establish and visualize the PPI network, and the cut-off criteria of interaction score was set as 0.4 (Szklarczyk et al., 2019). In the PPI network, the cytoHubba algorithm was utilized to identify the hub genes.

### Functional enrichment analyses of shared genes

GO enrichment analysis was utilized to explore the molecular function, cellular component, and biological process based on the shared genes. To predict the signaling pathways involved in diseases development, the KEGG pathway enrichment analysis was conducted using “clusterProfiler” R package (Yu et al., 2012). The *p*-value <0.05 was considered as the significant term.

### Identification and validation of prognostic signature

CC patients were randomly divided into the training group and the test group at a ratio of 1:1. Candidate prognostic genes were first selected in the training set through the univariate regression methods. Subsequently, LASSO penalty analysis was performed to avoid overfitting of the model. Furthermore, we applied multivariate regression to develop a prognostic signature. The risk factor was calculated as follows: $risk\;factor=\overset{n}{\underset{i=1}{\sum }}\left(\text{coef }\times {\text{Exp}}_{i}\right)$ . The Expi was the expression level of each gene and the coef was the risk coefficient of each gene. All patients were divided into high- and low-risk groups based on the median risk score, before the risk score of each patient being calculated.

### Verification of protein expressions of the hub genes

Human Protein Atlas (HPA) is an online tool utilizing transcriptomics and proteomics technologies to examine protein expression in different human tissues and organs at the RNA and protein levels. In the present research, we performed the HPA database to confirm the expression patterns of genes at the protein levels by immunohistochemistry.

### Gene set enrichment analysis

The gene expression data and risk groups information were enrolled into GSEA (Subramanian et al., 2005). A specific MSigDB v7.5 (released March 2020) database was further downloaded as the reference gene set. *p* < 0.05 and FDR <0.25 were considered as significant term to analyze enriched gene sets.

### Estimate of immune landscape

CIBERSORT is a powerful tool to characterize cell composition from complex tissues based on their gene expression data (Newman et al., 2015). CIBERSORT algorithms was utilized to depict the relative abundance of 22 types of immunocytes. *p* < 0.05 was selected as the threshold.

### Statistical analysis

All statistical data was analyzed by R version 4.0.5. The Kaplan–Meier survival analysis was next performed to compare the discrepancy in clinical outcomes of CC patients between two risk groups. ROC was employed to evaluate the predictive efficacy of the model. Univariate and multivariate Cox analyses were used to evaluate the independence of the model.

## Results

### Co-expression modules analysis

**FIGURE 1:**
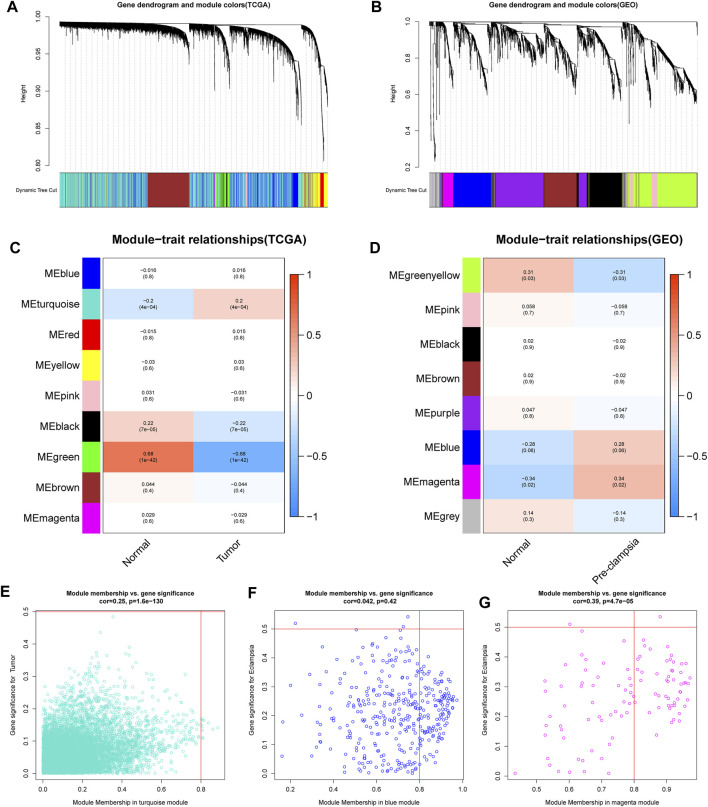
determination of the specific modules in PE and CC by WGCNA. **(A,B)** Cluster plots of co-expressed genes in two diseases. **(C,D)** Heatmap of module–trait in two diseases. **(E)** The “turquoise” model was selected as cervical cancer-related module. **(F,G)** The “blue” and “magenta” modules were selected as pre-eclampsia-related modules.

We first conducted WGCNA to obtain disease-specific modules with different colors (Figures 1A,B). In the in TCGA dataset, a total of 9 modules were determined and the “turquoise” model was selected as CC present-related module due to its high positive correlation with tumor trait (Figures 1C,E). In terms of the GSE60438 set, we uncovered 8 modules and choose “blue” and “magenta” modules as pre-eclampsia-related modules (Figures 1D,F,G).

### Identification of the shared genes in cervical cancer and pre-eclampsia

**FIGURE 2:**
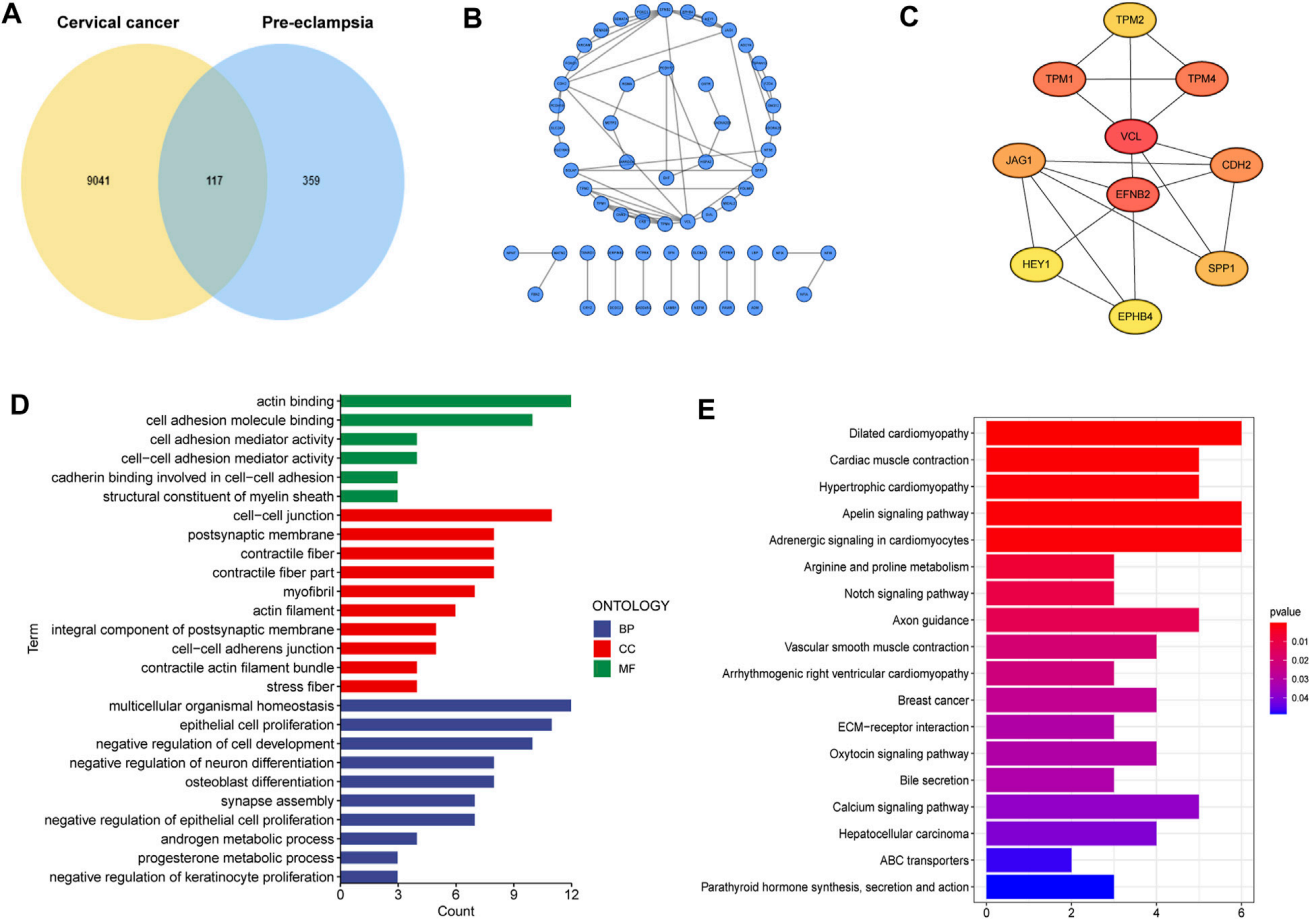
Characterization of the shared genes in PE and CC. **(A)** The Venn plot of overlapped genes. **(B)** The PPI network of the overlapped genes. **(C)** The top 10 hub genes of the PPI network. **(D)** GO function enrichment analysis. **(E)** KEGG enrichment analysis.

A total of 117 shared genes in CC and PE were extracted from three disease-specific modules, which may be greatly involved in the development of CC and PE (Figure 2A). To achieve a better understanding of the interrelationship of these shared genes, we create a PPI network by STRING online tool (Figure 2B). Moreover, we obtained 10 hub genes (VCL, EFNB2, TPM1, TPM2, TPM4, CDH2, JAG1, SPP1, HEY1 and EPHB4) with high MCC values according to the cytoHubba algorithm (Figure 2C).

Next, GO analysis was applied to unearth the underlying biological roles of above shared genes in two diseases. As revealed by Figure 2D, these genes were involved in numerous functions such as epidermal cells proliferation, regulation of cell development and neuron differentiation. In addition, KEGG enrichment indicated that the shared genes were activated in cardiac-related pathways, Notch signaling and ECM-receptor interaction (Figure 2E).

### Prognostic model development

**FIGURE 3:**
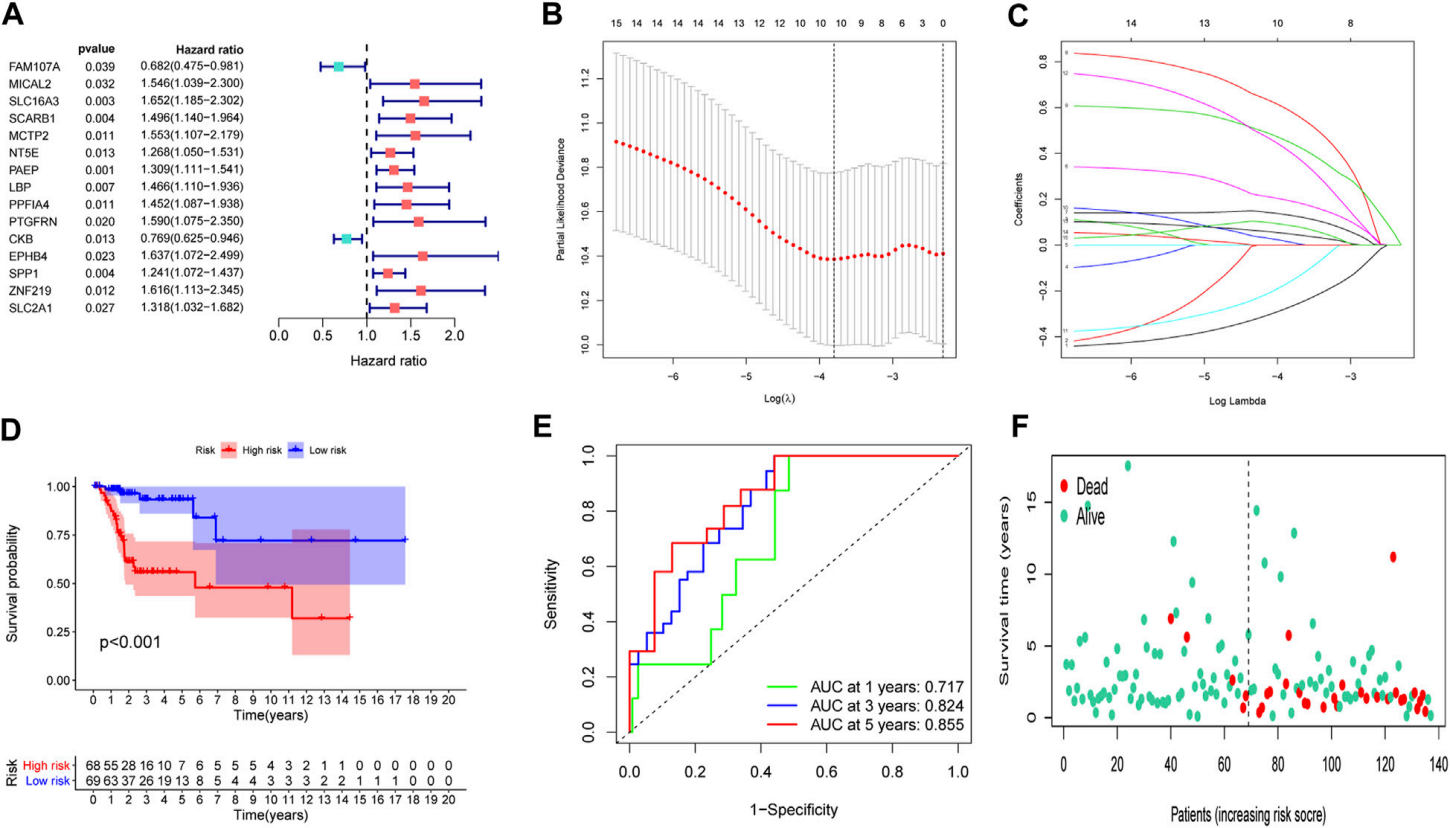
Construction of a prognostic model. **(A)** Univariate Cox regression analysis. **(B,C)** LASSO coefficients for model. **(D)** Survival analysis in the training set. **(E)** ROC curves of the predictive performance of the model in the training set. **(F)** The distribution of survival status in the training set.

To set up an optimal signature, all CC cases were randomly divided into training and test sets. In the training set, univariate Cox regression was employed to detect possible prognostic factors based on shared genes in two diseases (Figure 3A). A total of 15 prognostic genes from univariate analysis were then enrolled into LASSO regression (Figures 3B,C). Finally, we collected 10 genes (FAM107A, NT5E, PAEP, LBP, PPFIA4, PTGFRN, CKB, EPHB4, SPP1 and SLC2A1) to create a shared genes-based model by multivariate Cox method. Risk score = (-0.2665 × FAM107A) + (0.1905 × NT5E) + (0.1281 × PAEP) + (0.5798 × LBP) + (0.4434 × PPFIA4) + (0.0103 × PTGFRN) + (-0.1552 × CKB) + (0.3996 × EPHB4) + (0.0468 × SPP1) + (0.0834 × SLC2A1).

**FIGURE 4:**
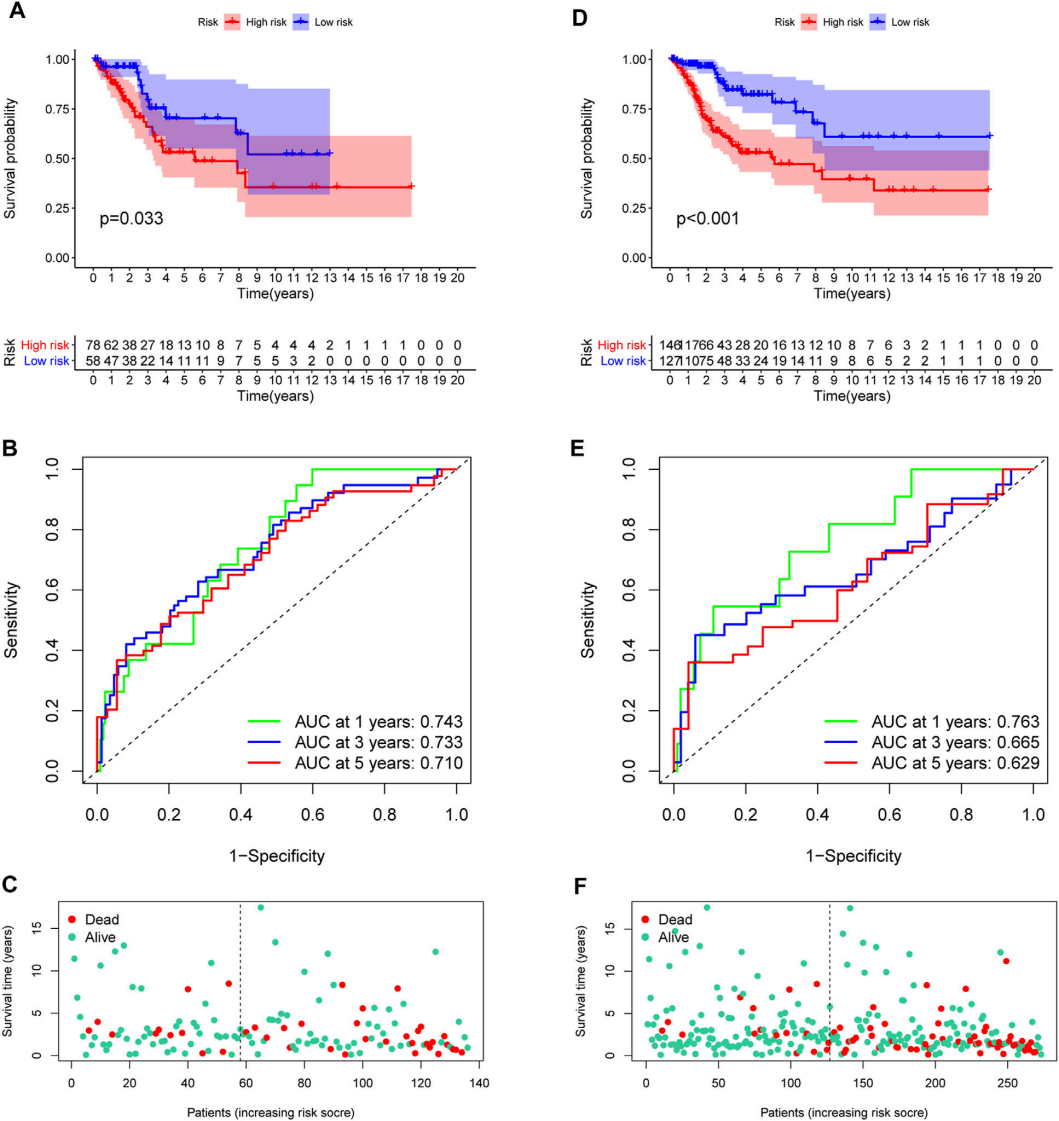
Validation of the prognostic model. **(A,D)** Survival analysis in the test and the entire cohorts. **(B,E)** ROC curves of the prognostic model. **(C,F)** The distribution of survival status in two verification sets.

In the training set, each CC sample was assigned a corresponding risk score and all patients were divided into high and low risk group based on the median risk score. Survival curves showed that overall survival (OS) of patients in the high-risk group was lower than that of patients in the low-risk group (Figure 3D). The AUCs for 1, 3 and 5-years survival rate were 0.717, 0824 and 0.855, respectively (Figure 3E). The risk plots of survival status were illustrated in Figure 3F. Then we confirmed the performance of the model in the test set and entire set according to the same analyses and observed the similar results. KM analysis indicated that the clinical outcome of the high-risk group was dismal than that in the low-risk group among the test and entired cohorts (Figures 4A,D). ROC analysis suggested that the AUCs of OS for 5-years survival rate were 0.710 and 0.629 in the test and entired cohorts, respectively (Figures 4B,E).

### Validation of the hub model genes

**FIGURE 5:**
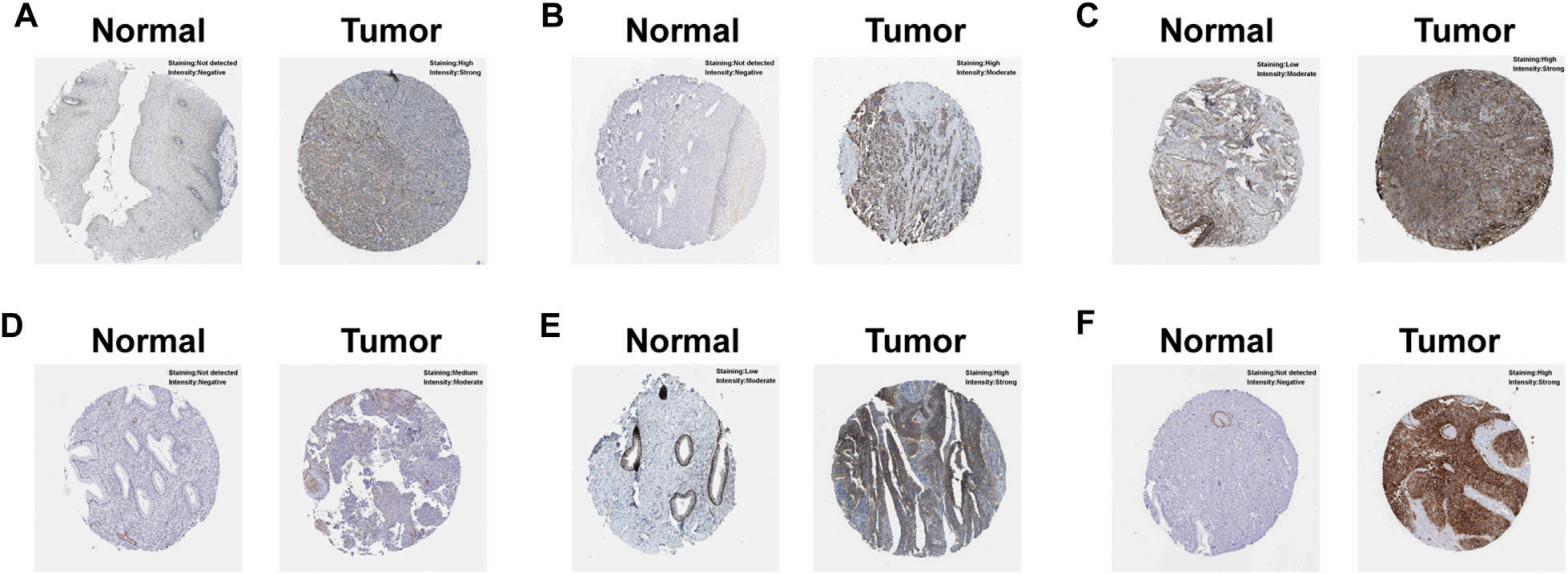
Verification of the hub model genes at protein level by HPA database. **(A)** EPHB4. **(B)** LBP. **(C)** NT5E. **(D)** PPFIA4. **(E)** PTGFRN. **(F)** SLC2A1.

In order to uncover the expression patterns of model factors at protein level, we conducted HPA tool. The results revealed that the protein levels of six model genes (NT5E, LBP, PPFIA4, PTGFRN, EPHB4 and SLC2A1) were greatly higher in CC tissues compared with normal tissues (Figure 5).

### Independent prognostic analysis

**FIGURE 6:**
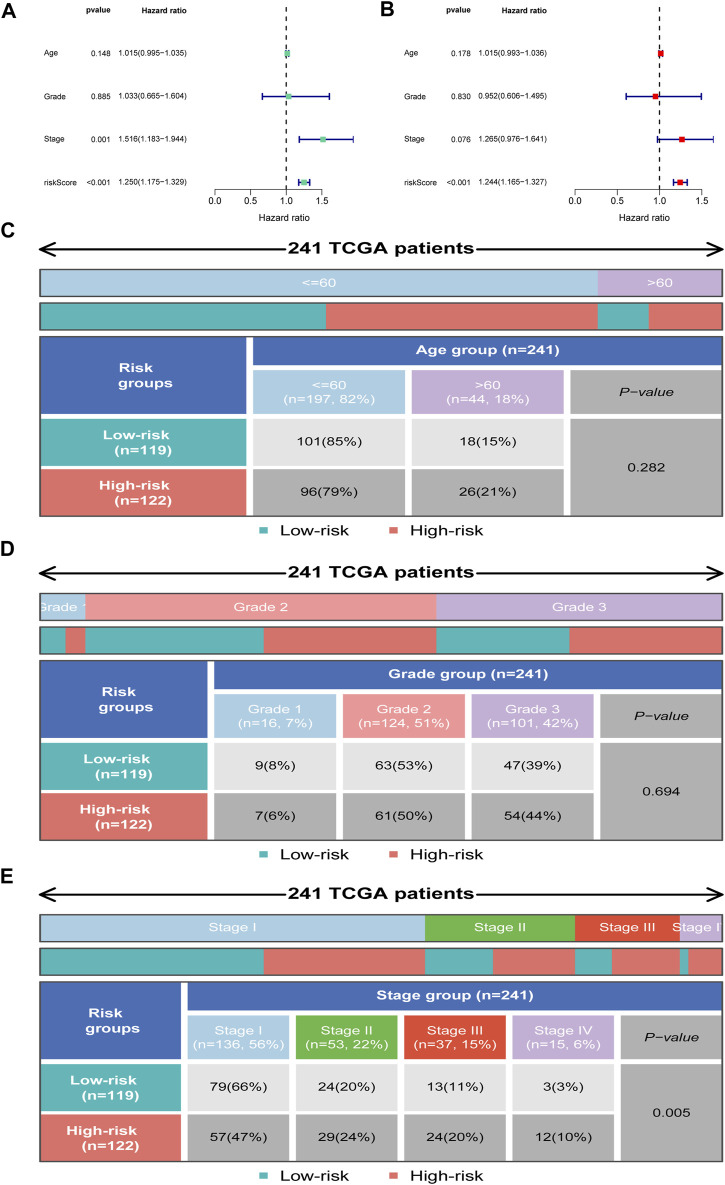
Independent prognosis analysis. **(A)** Univariate Cox regression analysis. **(B)** Multivariate Cox regression analysis. **(C–E)** Distribution of clinical subgroups in two risk groups.

We further employed univariate and multivariate Cox analyses to examine the independence of the signature. Univariate analysis showed that stage (*p* = 0.001) and the risk score (*p* < 0.001) were meaningful for assessing clinical outcome (Figure 6A). Multivariate Cox analysis showed that risk score (*p* < 0.001) was independent risk factors for prognosis evaluation in CC (Figure 6B). Additionally, we found that the two risk groups were remarkably correlated with four subgroups of stage, but no significant relationship with age and grade (Figures 6C–E).

### Subgroup analysis of the signature

**FIGURE 7:**
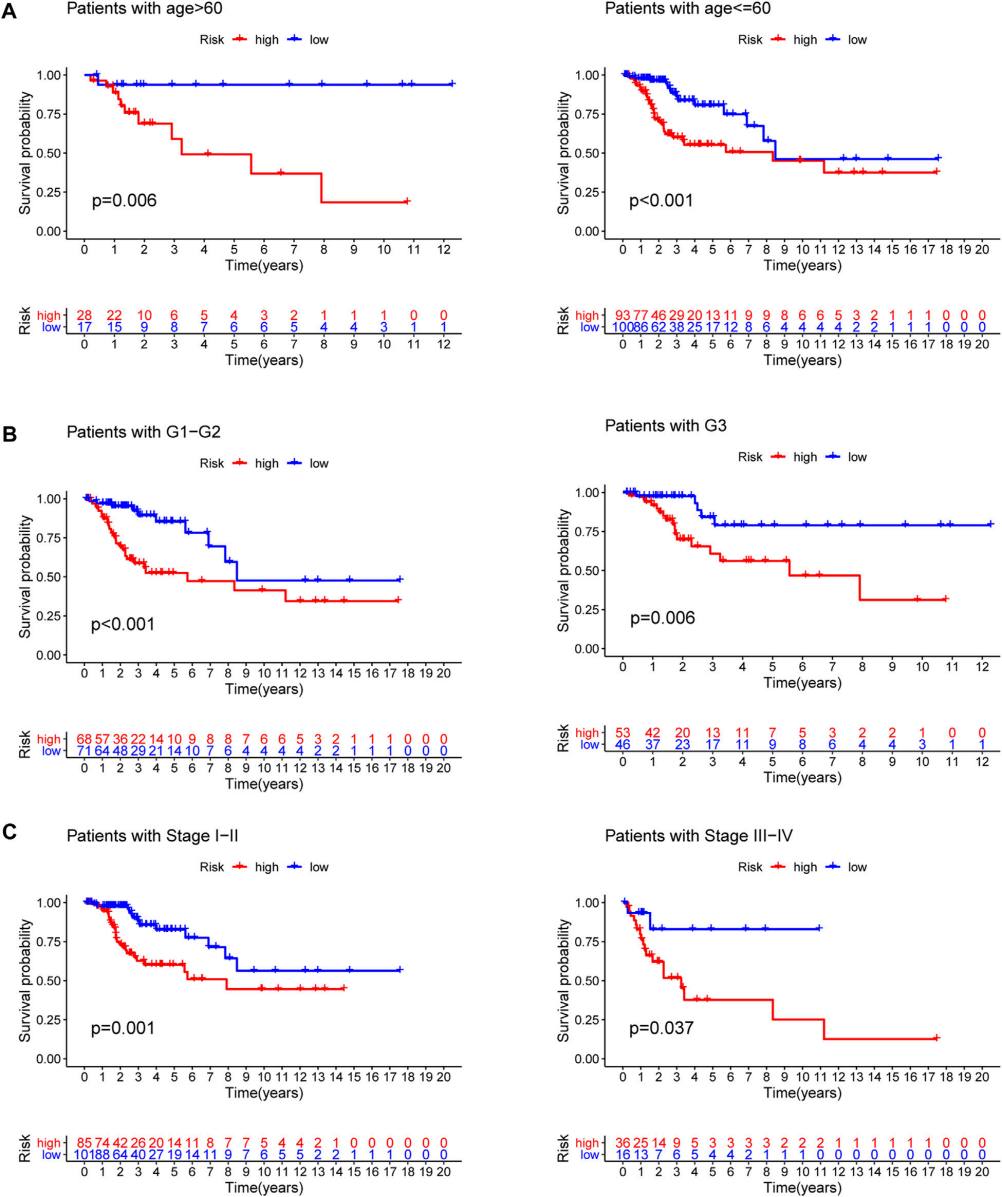
Subgroup survival analysis. **(A)** Age subgroup. **(B)** Grade subgroup. **(C)** Stage subgroup.

To explore the predictive value of the model in different subgroups of CC, all patients were categorized into three subgroups (age, grade and stage). The results of subgroup analysis suggested that high-risk group presented a dismal outcome compared to low-risk group based on three subgroups (Figure 7).

### Gene set enrichment analysis

**FIGURE 8:**
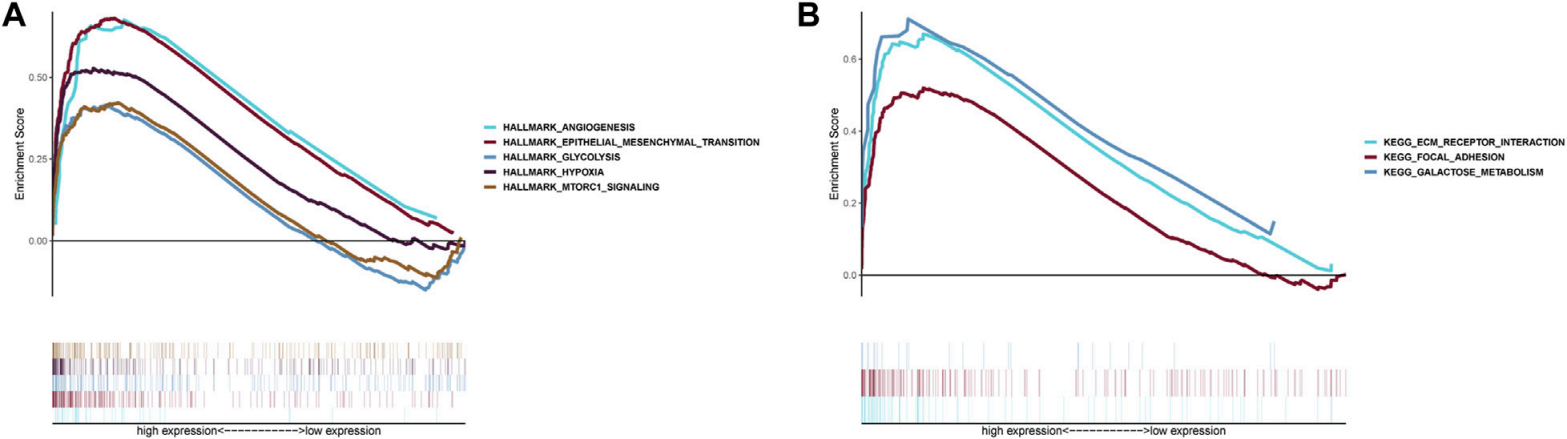
Gene Set Enrichment Analysis. **(A)** Hallmark analysis of the two risk groups. **(B)** KEGG analysis of the two risk groups.

GSEA indicated that five Hallmarks of CC were enriched in high-risk group, including “angiogenesis”, “epithelial-mesenchymal transition”, “glycolysis”, “hypoxia”, and “MTORC1 pathway” (Figure 8A). KEGG analysis suggested patients with high-risk were involved in ECM receptor interaction, focal adhesion and galactose metabolism (Figure 8B).

### Immunocyte infiltration analysis

**FIGURE 9:**
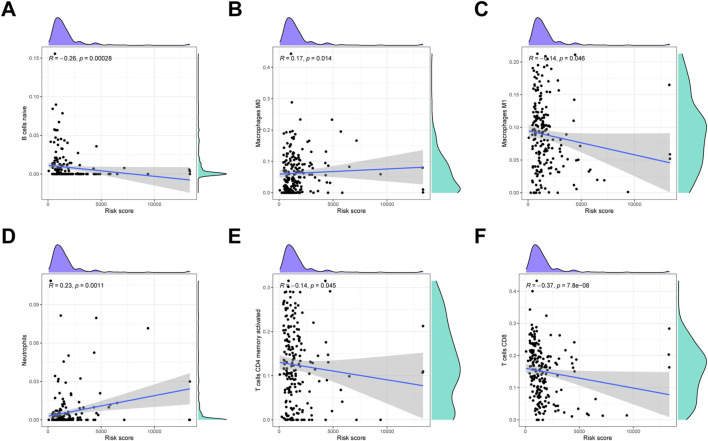
Immune infiltration analysis. **(A)** Naïve B cells. **(B)** Macrophages M0. **(C)** Macrophages M1. **(D)** Neutrophils. **(E)** Activated CD4 memory T cells. **(F)** CD8 T cells.

To detect the immune cells infiltration status of two groups, CIBERSORT algorithms was conducted to assess the proportion of various immunocytes. The results revealed that naïve B cells, macrophages M1, CD4 memory T cells and CD8 T cells downregulated in low-risk cohort, whereas macrophages M0 and neutrophils were enriched in high-risk cohort (Figure 9).

### Establishment of the RF model for PE

**FIGURE 10:**
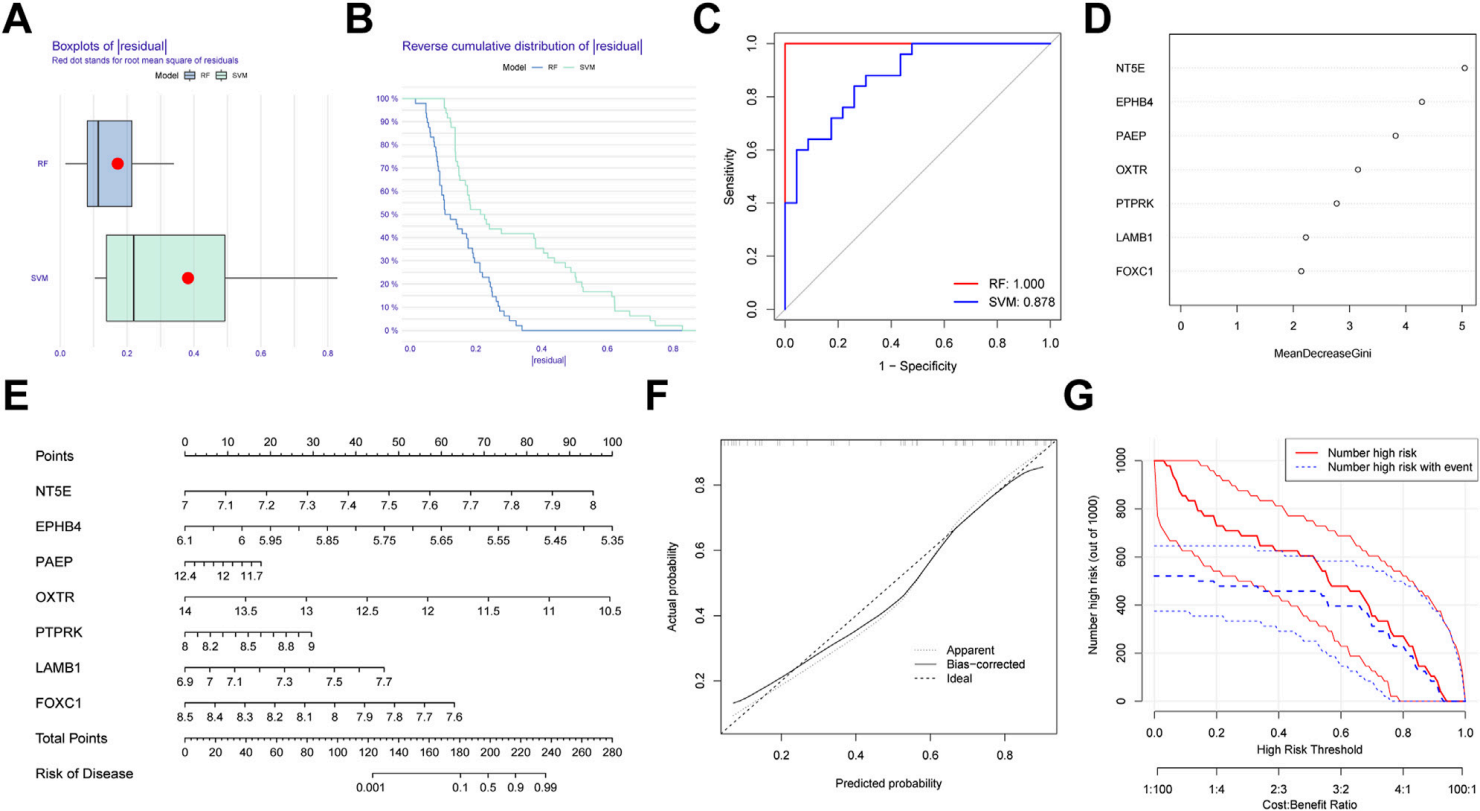
Establishment of the random forest (RF) Model for pre-eclampsia. **(A)** Boxplots of residual for RF and SVM model. **(B)** Reverse cumulative distribution RF and SVM model. **(C)** ROC curves indicated the accuracy of two models. **(D)** The importance of the seven hub genes based on the RF model. **(E)** Construction of a nomogram based on RF model. **(F)** Calibration curve revealed the predictive ability of the nomogram. **(G)** Clinical impact curve of the nomogram.

We first generated an RF and SVM model to collect potential indicator from the shared genes to predict the occurrence of PE. To determine the optimal PE model, the residuals of RF and SVM model were compared. We observed that the RF model has minimal residuals, suggesting it could be served as the favorable model to forecast the occurrence of PE (Figures 10A,B). Similarly, ROC curve showed the RF model has higher accuracy than SVM model (Figure 10C). Then, we ranked seven candidate genes based on their importance (Figure 10D).

In order to expend the performance of the RF model, a nomogram was constructed according to the seven potential genes to evaluate the prevalence of PE (Figure 10E). Calibration curve revealed the favorable ability of the nomogram (Figure 10F). Also, the clinical impact plot unearthed that the forecasting value of the nomogram was notable (Figure 10G).

## Discussion

Despite current preventative, screening, and treatment techniques, CC continues to pose a serious burden on public health for decades (Arbyn et al., 2020). According to the cancer incidence GLOBOCAN 2020 database, CC results in approximately 341,000 new deaths annually, and of note, more than 90% death from CC occurred in low- and middle-income countries, where effective screening and early diagnosis are relatively lacked (Siegel et al., 2018). Therapeutic options, as well as the survival rate of CC patients should benefit from a comprehensive understanding of the pathogenesis of CC.

The incidence of gynecological malignant cancers in pregnant women is increasing, among which, CC is the most frequently diagnosed cancer during pregnancy (Smith et al., 2003; Song et al., 2021b). Considering that gynecological screening is relatively bounded during pregnancy, efforts have been made to explore an effective prognostic model to guide the treatment for CC (McIntyre-Seltman and Lesnock, 2008). PE is a multisystem pregnancy complication characterized by cardiovascular dysfunction, with placental debris substantially released into the maternal circulation. As indicated earlier, there might exist an underlying correlation between the pathogenesis of CC and PE (Serrand et al., 2021). After that, a PPI regulatory network containing 10 hub genes was constructed based on these shared genes. It is worth noting that only limited information is available regarding the properties of most of genes in our regulatory network. For instance, as a core gene in the PPI network, VCL has previously been recognized as an independent factor in predicting the CC prognosis, while its role in PE development has yet been reported before (Sun et al., 2016; You et al., 2021). Further study should focus on validating the biological functions of these genes, as well as their roles in modulating the disease development.

Researches have been conducted to unveil the signaling pathways involved in facilitating development of CC and PE. According to our data, a group of molecular signalings including Epithelial-mesenchymal transition (EMT), hypoxia, mTOR, and glycolysis were identified to play vital roles in regulating the development of both CC and PE. EMT is one of the key steps of the metastatic cascade (Zeng et al., 2019). During EMT process, the loss of epithelial polarization induces multiple phenotypic changes and help cancer cells gain mesenchymal properties to disseminate and migrate quickly (Mathias et al., 2013). Hypoxia is well known as an independent prognostic indicator that is related to unsatisfactory treatment response and subsequent poor clinical outcome for CC patients (Mayer et al., 2013). Imai and his colleagues previously provided the evidence that hypoxia brought proteome changes are directly involved in the acquisition of metastasis behavior by activating EMT pathway, indicating that targeting hypoxia induced EMT may act as a promising strategy to prevent tumor invasion in CC (Imai et al., 2003). The PI3K/AKT/mTOR signaling pathway has been reported to regulate a series of cellular behaviors including cell proliferation, migration, and apoptosis in cancer cells (Saba et al., 2021). Regarding the pathogenesis of CC, researchers have pointed out that the aberrant activity of mTOR signaling plays a crucial role in regulating the crosstalk between HPV virus and host cells (Hoppe-Seyler et al., 2017). As has been pointed out before, the PI3K/AKT/mTOR mediated transcription repression of the viral E6/E7 oncogene can only be observed in hypoxia condition, indicating the connection between the PI3K/AKT/mTOR activity and metabolic transition in cancer cells (Bossler et al., 2019). Energy metabolism is gaining increasing attention in cancer cells recently. Cancer cells mainly rely on glycolysis to obtain sufficient energy regardless of the presence of oxygen, which is called the Warburg effect (Siska et al., 2020). Accumulating evidence shows that E6/E7 oncogene is responsible for the metabolic alteration in CC. In principle, E6 induces the degradation of p53, which in turn promotes glycolysis and restrains the oxidative phosphorylation (OXPHOS) pathways (Itahana and Itahana, 2018). It is worth noting that PI3K/AKT/mTOR cascades and hypoxia signaling are both involved in the glycolytic switch. As a metabolic sensor, mTOR complex responds to nutrient and energy production and render the accumulation of hypoxia-inducible factor 1 (HIF1) (Spangle and Munger, 2010). Other hypoxia related proteins are also found to exert pro-oncogenic effects by remodeling in glucose metabolism pattern. For instance, the hypoxia induced signal transducer and activator 5A (STAT5A) was proven to promote tumor cells growth by interrupting the activity of pyruvate dehydrogenase complex, a gatekeeper enzyme connecting glycolysis and the OXPHOS pathways (Zhang et al., 2021).

Establishment of immune landscape is essential for unveiling intricate relationships among clinical outcome and immune characteristics (Turinetto et al., 2022). It is well documented that recruitment of immunosuppressive cells protects cancer cells from surveillance by effector cells, which nullifies the immunotherapy and consequently promote cancer progression (Kitamura et al., 2015). It has been reported that M1-like macrophages is capable of killing tumor cells by initiating pro-inflammatory pathways within the TME (Mills et al., 2000). Likewise, it has been well established that cytotoxic CD8^+^ T cells are the most powerful effector cells in the adaptive immune system (Dustin, 2014). Cytotoxic CD8^+^ T cells are major killers of pathogens and neoplastic cells in TME (Farhood et al., 2019). Briefly, CD8^+^ T cells identify the MHC-1 molecules on the surface of antigen-presenting cells and neoplastic cells, and subsequently initiate the anticancer immune cascade (van der Leun et al., 2020). According to our data, the infiltration level of M1-like macrophage and anti-tumor CD8^+^ T cells is positively related to the improved clinical outcome for CC patients. In line with the common view that neutrophils can facilitate tumor proliferation by impairing the host immune system, the neutrophils infiltration is identified as immunosuppressive cells for being negatively correlated with OS of CC patients in our model (Shaul and Fridlender, 2019). Taken together, orchestration of immune characteristics may provide valuable evidence for the immunotherapy and help develop novel therapeutic targets against immunosuppressive environment.

However, there are still numerous shortcomings in our study. First, all data analyzed in this project were collected from public databases. The real world cohort need to be warranted to confirm the predictive ability of our model. In addition, experimental studies need to be conducted to explore the expression patterns and functional roles of the shared genes in CC in future work.

## Conclusion

The present project determined the shared genes to explore the pathogenesis of CC and PE and developed a shared genes-based signature which can be used as an indicator for clinical outcomes evaluation in CC.

## Data Availability

The original contributions presented in the study are included in the article/supplementary material, further inquiries can be directed to the corresponding authors.
