# Corrigendum: Exploration of shared gene signature with development of pre-eclampsia and cervical cancer

**DOI:** 10.3389/fgene.2022.1037320

**Published:** 2022-10-07

**Authors:** Tingting Yin, Yin Yin, Lin Qu

**Affiliations:** Department of Obstetrics and Gynecology, The First Affiliated Hospital of Nanjing Medical University, Nanjing, China

**Keywords:** pre-eclampsia, cervical cancer, tumor microenvironment, prognosis, WGCNA

In the published article, there was an error in the correspondence section, and correspondence author Lin Qu was erroneously excluded. The correspondence author list appears below: “Lin Qu, qulin1977@126.com; Yin Yin, yinyin20142747@njmu.edu.cn”

The authors apologize for this error and state that this does not change the scientific conclusions of the article in any way. The original article has been updated.

